# Research on the use of bedside ultrasound to quickly evaluate neurological prognosis in patients with ROSC

**DOI:** 10.1371/journal.pone.0358243

**Published:** 2026-09-16

**Authors:** Kerong Wang, Guoliang Li, Xiuci Yang, Meiling Ji, Shuainan Hong, Cong Liu, Mengyao An, Jiangning Yin

**Affiliations:** 1 Department of Emergency, The Affiliated Jiangning Hospital of Nanjing Medical University, Nanjing, China; 2 Nanjing Pukou People’s Hospital, Nanjing, China; Azienda Ospedaliero Universitaria Careggi, ITALY

## Abstract

**Objective:**

This study aims to find a quick bedside ultrasonography evaluation technique that evaluates the optic nerve sheath diameter to eyeball transverse diameter (ONSD/ETD) ratio and middle cerebral artery (MCA) flow parameters at 6 hours post-return of spontaneous circulation (ROSC) for early neurological prognosis.

**Methods:**

This retrospective study included 49 patients who achieved return of spontaneous circulation (ROSC) and were admitted to our emergency department between September 2023 and September 2025. Collected data encompassed baseline characteristics, GCS-M scores, APACHE-II scores, and bedside ultrasound measurements of ONSD, ONSD/ETD at 6, 24, and 48 hours post‑ROSC and middle cerebral artery hemodynamic parameters at 6, 24 hours post‑ROSC. The statistical analyses included the Mann-Whitney U test, t-test, and Receiver Operating Characteristic (ROC) curve analysis.

**Results:**

The ONSD/ETD ratio at 6 hours post-ROSC was a robust predictor of unfavorable neurological outcomes (AUC 0.922, *P* = 0.001), outperforming ONSD alone (AUC 0.866, *P* = 0.003), with an optimum threshold of 0.256 (sensitivity 76.5%, specificity 100%). The ONSD/ETD ratio was a significant predictor of CPC scores (OR = 1.979, *P* = 0.010). Although the majority of middle cerebral artery hemodynamic measures revealed no significant changes, all patients displaying cerebral circulatory arrest (CCA) waveforms or advancing to such waveforms demonstrated a dismal neurological prognosis. The alteration in Pulsatility Index (PI) was borderline significant (*P* = 0.05), with a threshold of 0.269 (AUC 0.90, sensitivity 80%, specificity 100%).

**Conclusion:**

The ONSD/ETD ratio assessed six hours after cardiac arrest accurately forecasts neurological prognosis, especially for unfavorable results (CPC 4–5). Transcranial Doppler can continuously monitor patients exhibiting an increased pulsatility index or identified cerebral circulatory arrest waveforms. The integration of various methodologies for dynamic evaluation enhances risk categorization and aids clinical decision-making for patients who attain ROSC. However, these findings are preliminary and require validation in larger cohorts.

## Introduction

Adverse neurological outcomes are prevalent in patients who achieve successful spontaneous circulation (ROSC) after cardiac arrest (CA) [1]. Enhancing clinical decision-making and reducing healthcare expenditures necessitate a timely and precise evaluation of neurological prognosis. Despite the reliability of multimodal prognostication in predicting outcomes, its extensive clinical use is constrained by its complex and costly implementation. Bedside ultrasound offers a viable solution, being a practical, cost-effective, and non-invasive method. Parameters such as cerebral blood flow and optic nerve sheath diameter (ONSD) are surrogate markers for assessing neurological prognosis, as they are closely associated with elevated intracranial pressure (ICP) levels [2]. Among TCD-derived parameters, end-diastolic velocity (EDV) is validated as sensitive indirect indicators for ICP assessment, because elevated ICP reduces cerebral perfusion pressure, markedly increasing diastolic cerebrovascular resistance—manifested as a progressive decline in EDV and a compensatory rise in pulsatility index [3].Elevated ICP also causes a synchronous pressure rise in the intraorbital subarachnoid space surrounding the optic nerve, which in turn expands the optic nerve sheath diameter [4]. Nevertheless, critical practical inquiries concerning the ideal scheduling for measurement, established cut-off values, and the specific functions of continuous versus single-time-point monitoring remain inadequately resolved for this ultrasound-based technique. This work seeks to systematically investigate the predictive usefulness of these characteristics and create a comprehensive, expedited evaluation process appropriate for emergency contexts.

## Subjects and Methods

### Research Participants

Clinical data from 49 patients who achieved return of spontaneous circulation (ROSC) and were admitted to the emergency medicine department of our hospital between September 2023 and September 2025 (data accessed on 21/11/2025) were collected for this retrospective investigation. Targeted temperature management (TTM) at 35–36°C was included in the standardized post-resuscitation therapy administered to all enrolled patients.

### Sample Size Consideration

Given the exploratory design and challenges in obtaining TCD windows after cardiac arrest, no a priori sample size calculation was performed. Nevertheless, a post-hoc power analysis for the primary endpoint (ONSD/ETD-6h) based on the observed effect size (Cohen’s d = 1.756) and group sizes (15 vs. 34) demonstrated that the achieved power exceeded 99.5% at a two-sided α = 0.05, confirming sufficient power to detect the large effect for the primary ultrasound parameter.

### Selection Criteria for Cases

- Individuals must be 18 years of age or older;
- Attainment of ROSC maintained for a minimum of 6 hours after cardiopulmonary resuscitation following in-hospital or out-of-hospital cardiac arrest is a prerequisites for inclusion.

### Exclusion criteria

Significant intracranial structural lesions (e.g., brain tumours, large-area cerebral infarction);

Conditions that may affect the measurement of ONSD or the evaluation of neurological prognosis (e.g., history of epilepsy, ocular defects, severe immune system diseases, psychiatric disorders, active thyroid eye disease, etc.).

### Observational Indicators

This study recorded patient baseline data in accordance with the Utstein style, including gender, age, height (H), weight (W), shoulder width (S), pupil distance (PD), and length of hospital stay (LOS). Both the APACHE-II score and the Glasgow Coma Scale motor component score (GCS-M) were recorded. The following were included in the cardiac arrest history: place of cardiac arrest (in-hospital cardiac arrest, IHCA, or out-of-hospital cardiac arrest, OHCA), duration of CPR (CPR time), neuron-specific enolase levels at admission (NSE-0h, NSE-48h), lactate level (Lac), pupillary light reflex, initial rhythm, and neurological reflexes following resuscitation.

The measurement instrument used was a Mindray M9pro (Model: UMT-500; S/N: 7E-28007844). All ultrasound measurements were performed by a single, trained emergency physician (who was not involved in the clinical decision-making process) using a standardized protocol. Data analysis was performed independently by another researcher.

### Methods of Measurement

**ONSD and ETD:** Select the image where the optic nerve is positioned at the center. The outermost border of the optic nerve sheath diameter (ONSDext), located 3 mm behind the globe at the junction with the ocular artery, and the eyeball transverse diameter (ETD) were measured using a linear array probe (5 MHz). Three measurements were made, and the average was calculated. The ONSD/ETD ratio was computed using the average value from both eyes.

**Cerebral Blood Flow:** The middle cerebral artery blood flow velocity was measured using a phased array probe (2 MHz) through the transtemporal window at a depth of roughly 5–7 cm. The end-diastolic velocity (EDV), mean velocity (MV), and peak systolic velocity (PSV) were all measured. The S/D ratio, resistive index (RI), and pulsatility index (PI) were computed.

To make sure there was no unilateral middle cerebral artery infarction or haemorrhage, all patients had CT or MRI scans. The patients had no prior history of vascular abnormalities or aneurysms. Measurements were made on the same side before and after. The blood pressure was kept above 90/60 mmHg while the readings were being taken.

### Outcome Metrics

Upon hospital discharge, the Glasgow-Pittsburgh Cerebral Performance Category (CPC) scale was employed to assess neurological prognosis. A favorable neurological outcome was denoted by a CPC score of 1–2, while an unfavorable neurological outcome was indicated by a score of 3–5.

### Analysis of Statistics

Statistical analysis was conducted using SPSS version 27.0. Post-hoc power analyses were conducted using PASS 15 (NCSS, LLC, Kaysville, UT, USA) to assess the power of the primary comparisons. The Shapiro-Wilk test was employed to confirm the normality of the measurement data. The independent samples t-test was employed for normally distributed data (*P* > 0.05), whilst the Mann-Whitney U test was utilized for non-normally distributed data (*P* < 0.05). Fisher’s exact test or the Chi-square test was employed to compare categorical data across groups. The diagnostic efficacy of relevant indicators and the optimal cut-off value (maximizing Youden’s index) were determined using Receiver Operating Characteristic (ROC) curves. A P-value below 0.05 was considered statistically significant, and all tests were conducted as two-tailed. The limitations of the small sample size were mitigated by the presentation of effect sizes for some indicators.

### Ethical approval

This retrospective observational study received approval from the institutional review board (Approval No.: 2025-03-095-K01) and was conducted in compliance with the ethical principles of the Declaration of Helsinki. In this retrospective investigation, the ethics committee has waived the requirement for informed consent. All personal identifiers were eliminated before data analysis to safeguard patient confidentiality. The authors’ access to patient data (from 2023 to November 2025) is permitted solely within the study period (September 2025 to January 2026). All access to identifiable participant information will be terminated after March 2026, and results are presented in aggregate to maintain anonymity.

## Results

### History of Cardiac Arrest and Baseline Characteristics of the Patient

This study comprised 49 patients who experienced return of spontaneous circulation after cardiac arrest. Upon discharge, the distribution of CPC scores was as follows: CPC 1 (n = 7, 14.3%), CPC 2 (n = 8, 16.3%), CPC 3 (n = 6, 12.2%), CPC 4 (n = 11, 22.4%), and CPC 5 (n = 17, 34.7%). Consequently, 69.4% (34/49) of patients experienced an unfavorable neurological outcome, while 30.6% (15/49) achieved a favorable neurological outcome. Statistically significant differences (*P* < 0.05) were identified between the excellent and poor outcome groups regarding the GCS motor score, APACHE II score, pupillary light reflex, duration of hospital stay, and location of cardiac arrest (in-hospital vs. out-of-hospital). Data are presented in Table 1.

**Table 1:** Basic information.

		Good prognosis	Poor prognosis	*t/U*	*P*	*Cohen d/* *Cramér’s V*
N	49	15	34			
APACHE-II		18.00 ± 6.78	25.06 ± 3.91	−2.879	0.015	−1.397
GCS-M		4.6 ± 1.0	2.0 ± 1.3	5.580	<0.001	2.140
weight (kg)		73.78 ± 31.12	67.12 ± 23.40	0.563	0.583	0.254
height (m)		1.71 ± 0.07	1.63 ± 0.21	1.564	0.133	0.494
shoulder (cm)		41.22 ± 3.53	39.18 ± 3.01	1.479	0.161	0.642
pupil diameter		2.9 ± 0.8	3.5 ± 1.6	−1.346	0.192	0.470
age (years)		60.11 ± 19.77	64.65 ± 17.95	−0.574	0.574	−0.244
NSE-6h		42.1 ± 15.8	20.2 ± 7.9	0.661	0.545	1.750
lac		8.5 ± 7.8	9.9 ± 4.0	−0.483	0.639	−0.226
gender (male)		13 (86.7)	24 (70.6)		0.380	0.207
Cause	Cardiogenic	10 (66.7)	17 (50.0)		0.075	0.475
	Pulmonary	4 (26.7)	8 (23.5)			
	Cerebral	1 (6.7)	9 (26.5)			
Pre-CPR time<4min		13 (86.7)	22 (64.7)		0.063	0.399
CPR time <30 min		13 (86.7)	22 (64.7)		0.063	0.399
Shockable Rhythm		8 (53.3)	6 (17.6)		0.470	0.028
IHCA		13 (86.7)	17 (50.0)		0.023	0.485
light reflex(+)		10 (66.7)	7 (20.6)		0.008	0.566
BMI (kg/m2)		22.04 (21.22‑23.66)	22.34 (20.76‑26.12)	68.5	0.682	
NSE-48h		24.7 (15.5–165.1)	20.8 (16.7–24.9)	3.0	0.800	
LOS(d)		9 (7‑15)	3 (1‑7)	30.5	0.011	
Pupil Distance (cm)		7.0 (6.5‑7.5)	7.0 (6.5‑7.0)	67.5	0.640	

“Shoulder” refers to the width of the shoulder. Pupil distance is defined as the linear distance between the centers of the two pupils when the eyes are in primary gaze. + denotes the presence of the pupillary light reflex.

#### Parameters for Bedside Ultrasound.

**Fig 2:**
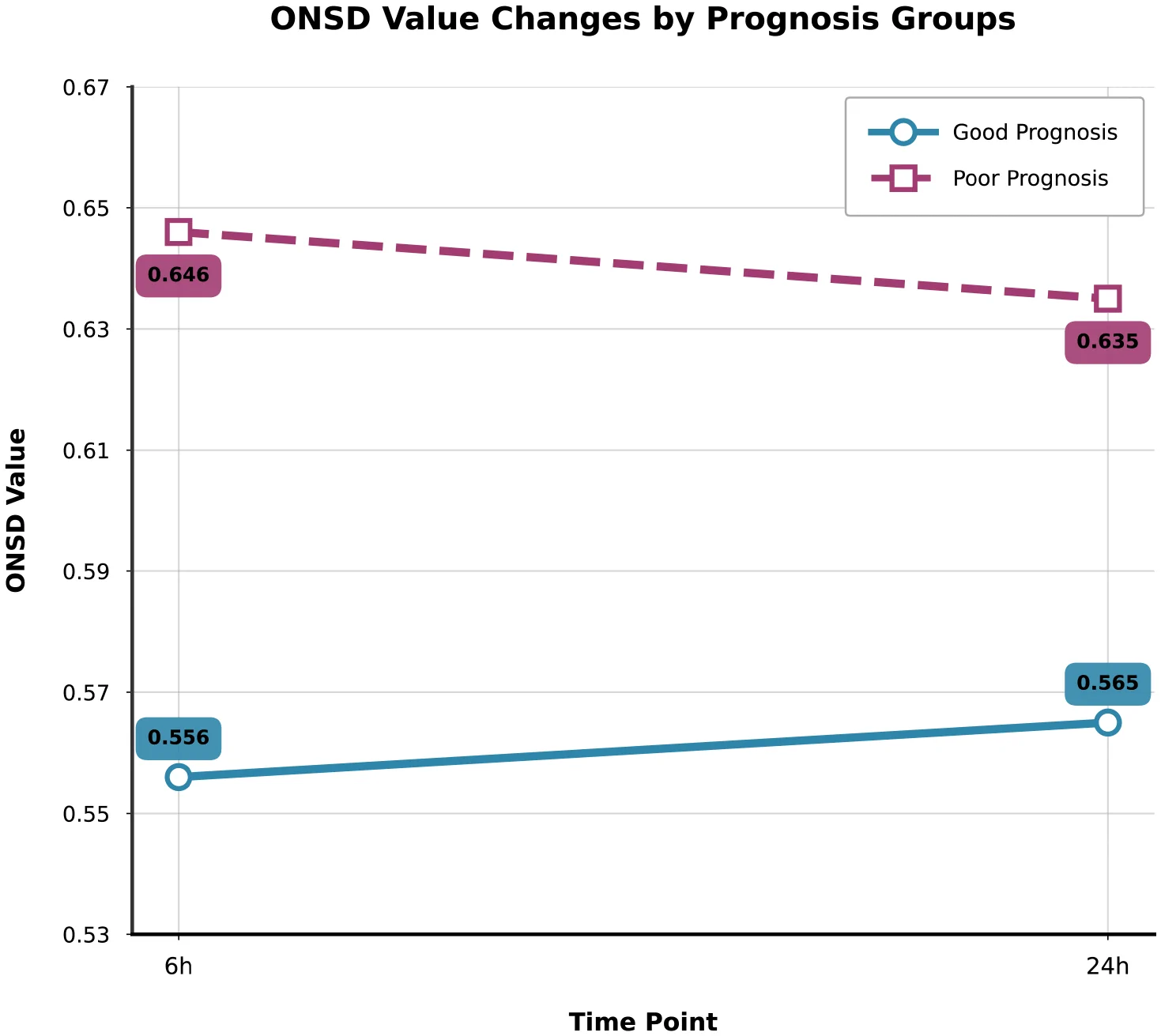
The average value change trend of ONSD.

#### Correlation between Adverse Outcomes and ONSD-Related Variables.

We analyzed ONSD and ONSD/ETD at 6, 24, and 48 hours post-ROSC, as well as the ratios of ONSD at 6 hours to several basic body parameters (refer to Table 2). The numbers of patients with available measurements were 49/49 at 6 hours, 26/49 at 24 hours, and 15/49 at 48 hours. In comparison to the excellent outcome group, the poor prognosis group’s ONSD-6h, ONSD/ETD-6h, ONSD/Height (O/H), ONSD/Pupil Distance (O/PD), and ONSD/Shoulder width (O/S) values were significantly elevated. In the poor prognosis group, ONSD-24h and ONSD/ETD-24h showed an increasing trend but no statistically significant difference.

**Table 2:** ONSD and related parameters measured by bedside ultrasound.

Varies	Good prognosis (n = 15)	Poor prognosis (n = 34)	*t*	*P*	*Cohen d*
ONSD/H(mm/m)	3.098 ± 0.403	3.929 ± 0.809	−3.497	0.002	−1.187
ONSD/W(mm/kg)	0.081 ± 0.030	0.103 ± 0.039	−1.642	0.116	−0.621
ONSD/BMI(mm*m2/kg)	0.233 ± 0.070	0.263 ± 0.065	−1.077	0.298	−0.454
ONSD/PD(mm/cm)	0.767 ± 0.140	0.919 ± 0.139	−2.637	0.018	−1.090
ONSD/S(mm/cm)	0.129 ± 0.019	0.160 ± 0.019	−3.966	0.001	−1.651
ONSD-6h(mm)	5.314 ± 0.462	6.216 ± 0.067	−3.535	0.002	−1.457
ONSD/ETD-6h(mm/mm)	0.235 ± 0.025	0.278 ± 0.029	−4.259	<0.001	−1.756
ONSD-24h	5.651 ± 0.416	6.348 ± 0.801	−1.929	0.093	−1.126
ONSD/ETD-24h	0.248 ± 0.017	0.280 ± 0.030	−2.291	0.052	−1.329
ONSD-48h	5.703 ± 0.622	5.921 ± 0.924	−0.301	0.800	−0.308
ONSD/ETD-48h	0.251 ± 0.020	0.265 ± 0.036	−0.520	0.677	−0.571

ONSD/H: the ratio of ONSD to patient height. ONSD/W: the ratio of the ONSD to patient weight. ONSD/BMI: the ratio of the ONSD to body mass index (BMI). ONSD/PD: the ratio of the ONSD to pupillary distance. ONSD/S: The ratio of ONSD to shoulder width. ONSD-6h, ONSD-24h, ONSD-48h: The ONSD measured at 6, 24, 48 hours after ROSC.

**Fig 1:**
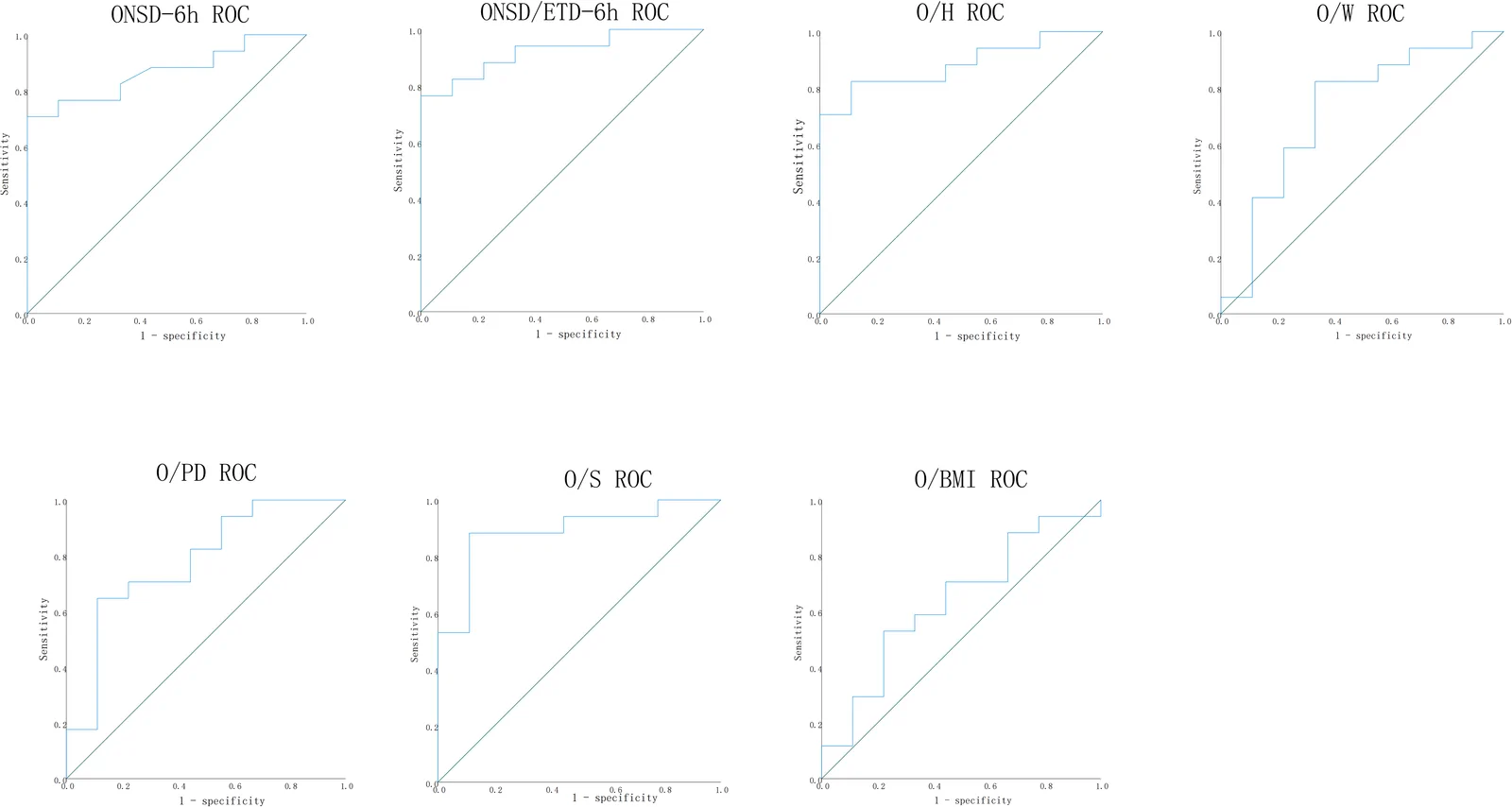
ROC curve of ONSD and related parameters.

The ONSD/ETD-6h ratio demonstrated the highest area under the curve (AUC) for forecasting an unfavorable result, at 0.922 (*P* < 0.001; 95% CI: 0.820–1.000), as per ROC analysis of the markedly distinct traits. The optimal cut-off value was 0.256, yielding a specificity of 100% and a sensitivity of 76.5%. In comparison to ONSD-6h alone, it exhibited superior predictive performance (AUC 0.866, *P* = 0.003; 95% CI: 0.728–1.000). Fig 1 illustrates the ROC curves, while Table 2, Table 3 delineates the corresponding parameters.

**Table 3:** ONSD and associated parameters ROC.

Variable	AUC	*P*	*95%CI*	Cut off value	sensitivity	specificity
ONSD/PD	0.778	0.022	0.581-0.974	0.891	64.7%	88.9%
ONSD/H	0.882	0.002	0.753-1.000	3.439	82.4%	88.9%
ONSD/S	0.889	0.001	0.756-1.000	0.146	88.2%	88.9%
ONSD-6h	0.866	0.003	0.728-1.000	5.833	70.6%	100.0%
ONSD/ETD-6h	0.922	0.001	0.820-1.000	0.256	76.5%	100.0%
ONSD-24h	0.714	0.199	0.411-1.000	6.791	33.3%	100.0%
ONSD/ETD-24h	0.810	0.063	0.537-1.000	0.278	66.7%	100.0%

^a^The optimal cut-off value was determined by maximizing Youden’s index

### Potential Association Between Prognosis and TCD

At 6 hours (19/49) and 24 hours (9/49) post-ROSC, we assessed PSV, EDV, MV, PI, RI, and S/D ratios for the 19 patients who successfully underwent TCD monitoring. We analyzed the absolute values of velocity parameters for spectra with consistent directions (14/19) and excluded spectra with discordant PSV and EDV directions (cerebral circulatory arrest waveforms) to ensure data integrity. Table 4 indicates that there were no statistically significant differences in any of the TCD characteristics. Irrespective of the elevation of the ONSD/ETD ratio, patients exhibiting cerebral circulatory arrest waveforms (5/19) during the monitoring interval promptly progressed to clinical brain death, as indicated by an analysis of the unilateral middle cerebral artery TCD spectra (Fisher’s exact test: *P* = 0.04, *Cramér’s V* = 0.51).

**Table 4:** TCD and prognosis.

Varies	Good prognosis	Poor prognosis	*t*	*P*	*Cohen d*
PSV-6h (cm/s)	61.45 ± 21.70	63.67 ± 34.95	−0.057	0.955	−0.079
EDV-6h (cm/s)	19.57 ± 9.13	23.63 ± 21.03	−0.452	0.656	−0.257
MV-6h (cm/s)	41.32 ± 11.92	43.78 ± 21.45	−0.212	0.834	−0.144
PI-6h	1.03 ± 0.39	0.93 ± 0.30	0.663	0.515	0.287
RI-6h	0.67 ± 0.16	0.67 ± 0.13	0.118	0.907	<0.001
S/D-6h	4.03 ± 2.80	3.42 ± 1.69	0.538	0.597	0.266
PSV-24h (cm/s)	69.40 ± 18.76	98.87 ± 38.96	−1.341	0.251	−0.969
EDV-24h (cm/s)	23.86 ± 14.12	32.92 ± 20.99	−0.649	0.552	−0.498
MV-24h (cm/s)	50.96 ± 12.99	63.77 ± 14.43	−1.107	0.330	−0.933
PI-24h	0.90 ± 0.19	1.03 ± 0.05	−0.894	0.422	−0.971
RI-24h	0.66 ± 0.15	0.68 ± 0.09	−0.166	0.877	−0.158
S/D-24h	3.50 ± 1.65	3.30 ± 0.92	0.157	0.883	0.149

### Trends in ONSD and TCD-Related Parameters in ROSC Patients and Their Potential Prognostic Association

**Fig 3:**
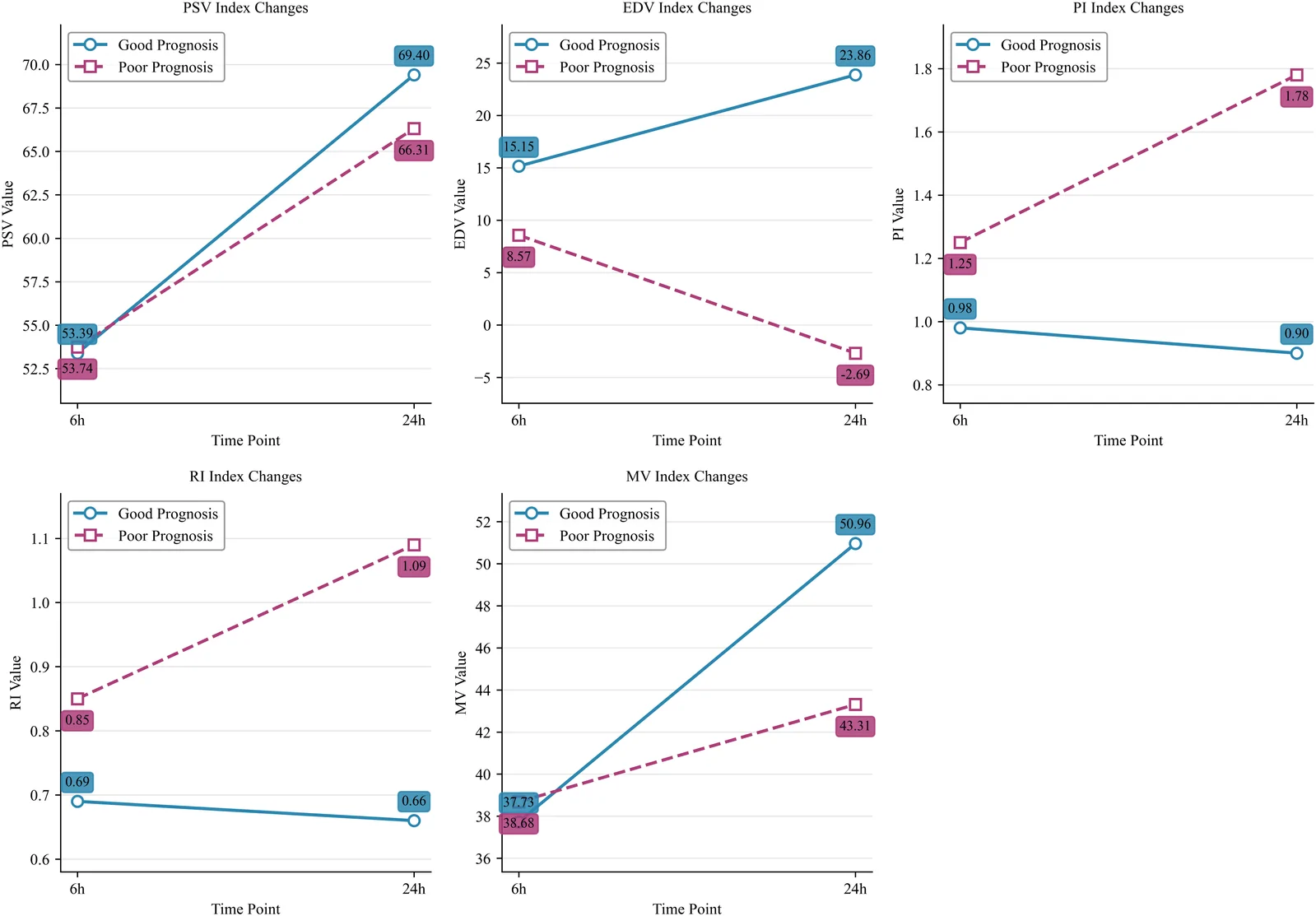
The average trend of changes in parameters of TCD.

This study investigated the relationship between cerebral blood flow parameters in nine patients (4/5) and prognosis, as well as the variations and trends in ONSD from six to twenty-four hours in 26 patients (13/13). The data demonstrated a decreasing trend in ONSD throughout a 24-hour period. PSV, EDV, and MV in the middle cerebral artery increased during 24 hours in patients with a favorable prognosis, but PI and RI exhibited a modest reduction. In contrast to the group with a favorable prognosis, the group with an unfavorable prognosis had increases in PSV and MV, though to a smaller extent. RI and PI exhibited large increases, while EDV had a considerable decline to negative levels. Fig 2, Fig 3. The intergroup variation in the alteration of Pulsatility Index (∆PI) was statistically significant (*P* = 0.05). The median ∆PI was markedly elevated in the poor prognosis group (0.559) relative to the excellent prognosis group (0.210), with a corresponding area under the ROC curve (AUC) of 0.90 (95% CI: 0.681–1.000). The sensitivity and specificity for forecasting a poor prognosis were 80% and 100%, respectively, with an optimal cut-off value of 0.269. No evident correlation existed between the prognosis and the patterns or directions of changes in any other metric. The results are presented in Table 5,Table 6.

**Table 5:** Changes in TCD Hemodynamic Parameters from 6 to 24 Hours and Prognosis.

	Good prognosis	Poor prognosis	*U*	*P*
ΔPSV	17.655（8.373–23.845）	27.493(14.690–30.630)	5	0.221
ΔEDV	8.772(3.410–15.843)	18.987(11.333–24.573)	6	0.327
ΔMV	13.557（6.112–20.348）	11.322(9.489–22.107)	8	0.624
ΔPI	0.210（0.159–0.260）	0.559(0.272–0.843)	2	0.050
ΔRI	0.102（0.085–0.139）	0.178(0.100–0.356)	5	0.221
ΔONSD	0.015 (0.002–0.023)	0.025(0.000–0.018)	14.5	0.352
ΔONSD/ETD	0.104 (0.000–0.025)	0.006(0.001–0.012)	18.000	0.667

Mann-Whitney U test was used for non-normally distributed data. Δ (delta) represents the absolute value of the difference between the 24-hour parameter and the 6-hour parameter (|value₂₄h – value₆h|).

**Table 6:** Direction of Hemodynamic Parameter Changes from 6 to 24 Hours and Prognosis.

Varies^a^	Good prognosis	Poor prognosis	*P*	Cramér’s V
ΔONSD(↓/ ↑ /—)	7/4/2	8/1/4	0.559	0.289
ΔPSV(↓/↑)	0/4	1/4	1.000	0.316
ΔEDV(↓/↑)	2/2	2/3	1.000	0.100
ΔMV(↓/↑)	4/0	3/2	0.444	0.478
ΔPI(↓/↑)	1/3	3/2	0.524	0.350
ΔRI(↓/↑)	1/3	3/2	0.524	0.350

A downward arrow (↓) denotes a decrease in the corresponding parameter at 24 hours compared to the 6-hour measurement; a hyphen (-) indicates no change, and an upward arrow (↑) signifies an increase.

### Ordinal Logistic Regression Analysis of ONSD/ETD and CPC Grade

This study evaluated the predictive impact of ONSD/ETD（Owing to the small values of the ONSD/ETD ratio, standardization was performed by multiplying by 100 to facilitate interpretation）on CPC grade by ordinal multinomial logistic regression analysis involving 49 ROSC patients. The model exhibited considerable explanatory capacity (Cox-Snell R² = 0.406, Nagelkerke R² = 0.426) and a satisfactory fit (likelihood ratio χ² = 25.556, *P* < 0.001). The results indicated that ONSD/ETD-6h exhibited enhanced predictive capability for CPC 4–5 and effectively distinguished between patients with CPC 1–2 and CPC 3–5. For each one-unit increase in ONSD/ETD‑6h, the odds of transitioning to a higher CPC grade increased by approximately 97.9% (OR = 1.979, 95% CI: 1.175–3.335, P = 0.010).

## Discussion

### ONSD/ETD at 6h after ROSC is a reliable indicator of Neurological Prognosis

Absolute ONSD values are influenced by individual factors, including ethnicity [5–9] and gender [10]. To correct for these inter-individual variations, the Vaiman group proposed adopting the ETD as an internal reference [11], with the ONSD/ETD ratio eliminating the confounding effect of eyeball size. CT-measured ONSD/ETD in healthy adults was 0.19 ± 0.02, compared with 0.29 in patients with non-traumatic intracerebral hemorrhage [12]. Kim DH [8] further confirmed the stability of this ratio on ultrasonography, demonstrating that it is independent of sex, height, BMI, and ethnicity. These converging lines of evidence indicate that the ONSD/ETD ratio effectively adjusts for inter-individual anatomical variability, possesses greater cross-population applicability than absolute ONSD, and represents a preferred metric for intracranial pressure assessment. Building on these advantages, previous research predicted the neurological prognosis of ROSC patients using CT-measured ONSD/ETD, yielding a ROC AUC of 0.66 (95% CI: 0.56–0.75), with an optimal cut-off value of 0.257, sensitivity of 85.7%, and specificity of 45.8% [13]. Our study demonstrates that ultrasound-derived ONSD/ETD, measured as early as 6 hours post-ROSC, enables early neurological prognostication. Notably, compared with both absolute ONSD and correction methods based on various body parameters, the ONSD/ETD ratio exhibited the optimal predictive performance, offering significantly superior discrimination between favorable and unfavorable outcomes and higher accuracy for identifying patients with CPC grades 4–5. This finding is consistent with prior evidence that ETD provides a robust correction for ONSD.

### ONSD and ONSD/ETD changes over 6–24h did not differ significantly between the two groups

Within 24 hours post-ROSC, the ONSD and ONSD/ETD ratio often demonstrated a little decline in the favorable prognosis group, whereas it displayed an increasing or consistently high trend in the unfavorable prognosis group. Nonetheless, neither group exhibited a statistically significant alteration in trend. This study cannot now demonstrate a definitive correlation between neurological outcome and short-term variations or trends in ONSD and ONSD/ETD. The discerned slight tendency indicates that during TTM, intracranial pressure may diminish in individuals with a favorable prognosis, whereas it may remain stable or escalate in those with an unfavorable prognosis.

### Cerebral circulatory arrest and ΔPI specifically forecast poor prognosis after ROSC

Previous studies generally consider PI and EDV to be important indicators reflecting ICP changes [14,15]. However, in this study, none of the indicators exhibited a significant association with neurological outcome. This conclusion may arise from the intricacy of cerebrovascular regulatory mechanisms in patients with ROSC. Cerebral blood flow velocity is regulated by various pathophysiological factors such as partial pressure of carbon dioxide, hematocrit [16], and body temperature [17]. During the initial phases of high intracranial pressure, cerebral blood vessels compensatorily augment blood pressure and sustain cerebral perfusion via autoregulation, potentially stabilizing blood flow velocity characteristics for an extended duration. Only when intracranial pressure is consistently elevated and advances to the decompensated stage does a reduction in end-**diastolic** flow velocity or a notable increase in pulsatility index become evident. This study revealed that a significant increase in the PI, accompanied by a decrease or reversal of EDV—characteristics associated with cerebral circulatory arrest waveforms (e.g., spike or oscillating waves)—collectively signify cerebral circulatory decompensation and act as sensitive early predictors of unfavorable neurological outcomes. The amplitude of change in PI was more pronounced in individuals with poorer prognosis than in those with better prognosis, indicating that patients with poor prognosis may experience higher oscillations in intracranial pressure. Although the change in PI reached borderline significance (*P*  =  0.05), which should be interpreted with caution, the presence of CCA waveforms in 5 patients served as a definitive clinical marker of irreversible brain damage. This qualitative finding is more robust than the quantitative PI change in this small subset. All patients exhibiting CCA waveforms [14] in the unilateral middle cerebral artery swiftly advanced to brain death, thus affirming the great specificity of this spectral pattern in predicting an exceedingly unfavorable prognosis. This finding suggests that TCD-detected spectral waveform changes may be of greater prognostic value than isolated numerical changes in the neurological outcome assessment of patients with ROSC.

### Clinical Implication

**Fig 4:**
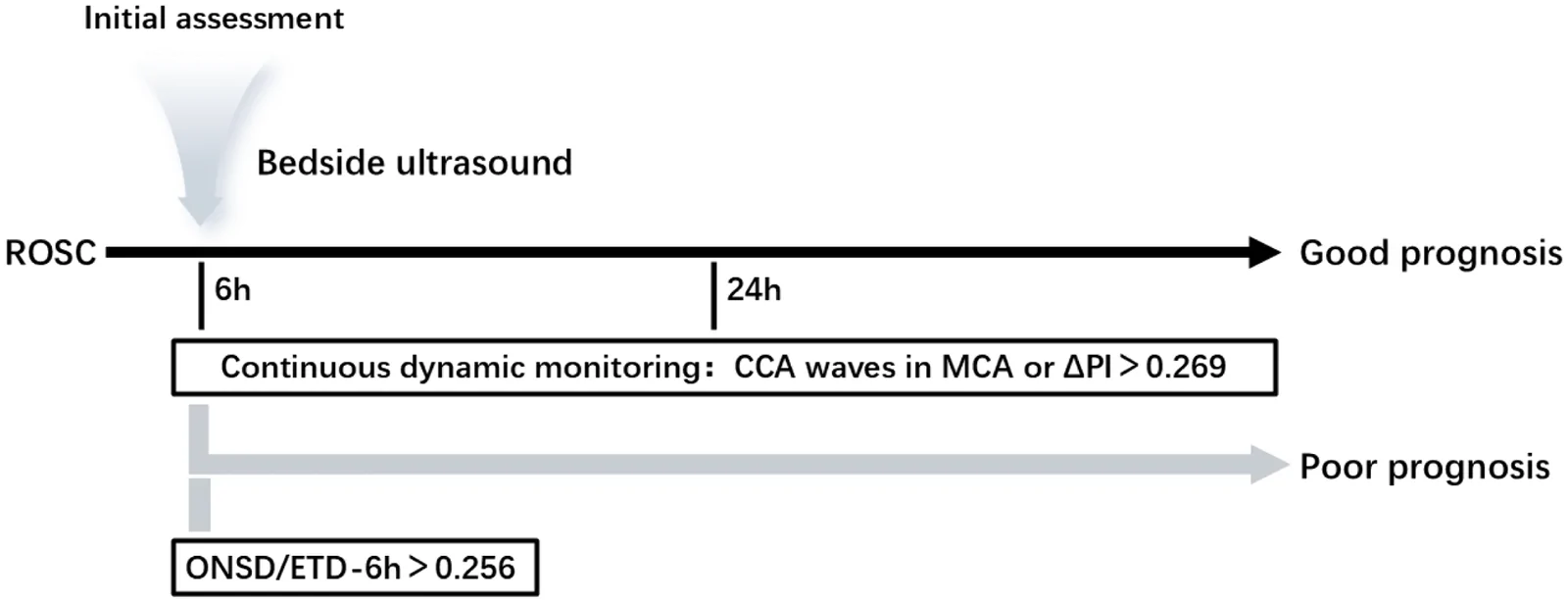
Clinical practice process.

Based on the findings of this study, a potential bedside ultrasound assessment strategy may be outlined as follows. Our results suggest that it may be reasonable to perform TCD and ocular ultrasound examinations at 6 and 24 hours post‑ROSC, with continued monitoring every 24 hours if resources permit. Because short-term fluctuations in ONSD were not clearly associated with prognosis in our analysis, we found the ONSD/ETD ratio at the 6-hour time point to be more informative, and continued TCD monitoring may provide complementary information. In this cohort, patients with an ONSD/ETD‑6h ratio ≥ 0.256 were more likely to experience unfavorable neurological outcomes, suggesting that this threshold could help identify individuals at higher risk at an early stage, while those with values below 0.256 were more frequently associated with better outcomes but still required close dynamic observation. Notably, the detection of a unilateral cerebral circulatory arrest waveform on TCD appeared to be an ominous sign irrespective of the ONSD/ETD-based stratification, as it was uniformly associated with poor prognosis in our series. Furthermore, among patients under ongoing observation, a ΔPI increase greater than 0.269 within 24 hours may indicate an elevated risk of poor outcome and should be interpreted together with clinical findings and other signs. A flowchart summarizing these observations is presented in Fig 4.

### Limitation

This study has several limitations that should be acknowledged. First, as a single‑center retrospective analysis, the overall sample size was modest, although post‑hoc power analysis for the primary endpoint (ONSD/ETD at 6h) was adequate (>99.5%). Second, data attrition occurred at later time points (24 h and 48 h), and the sample available for TCD analysis was notably smaller—particularly for longitudinal assessments (from 49 to 19 patients at 6h, and to 9 for dynamic analyses)—which affected the statistical power of these exploratory TCD parameters (e.g., post‑hoc power for ΔPI was approximately 22%). Third, TCD measurements were obtained unilaterally owing to operational constraints, which may have limited the comprehensiveness of cerebral flow assessment. Fourth, the predictive model lacks external validation, and no medium‑ or long‑term follow‑up data were available for neurological outcomes. Despite these considerations, the primary ultrasound parameter demonstrated robust prognostic performance, and the TCD findings, while preliminary, provide valuable hypotheses for future investigation. Accordingly, multi‑center prospective studies with larger cohorts and standardized longitudinal protocols are warranted to confirm and extend these observations.

## Conclusions

This study illustrates that a swift bedside ultrasonography approach is an effective alternative for early neuroprognostication. The ONSD/ETD ratio at 6 hours post-resuscitation is a robust predictor of unfavorable neurological outcomes. TCD detection of cerebral circulatory arrest waveforms is a highly specific marker of poor prognosis, whereas considerable PI fluctuation suggests a poorer prognosis. This accessible, non-invasive method serves as a vital decision-support tool in the critical window before MRI becomes available, allowing clinicians to promptly identify high-risk patients and customize therapy strategies accordingly.

## Supporting information

S1 File Raw data. (PDF)
