# Supplementary material for: Research on the use of bedside ultrasound to quickly evaluate neurological prognosis in patients with ROSC
Source: PLoS One. 2026 Sep 16;21(9):e0358243. doi: 10.1371/journal.pone.0358243 (PMC13581026; doi:10.1371/journal.pone.0358243)
Supplement: Supplementary file 1 [file pone.0358243.s001.pdf]

ID	male=1 female=2	good=1-2；POOR=3-5	0=good；1=poor	year	Pupil Distancecm	cm	heightm	weightkg	BMI
1	2	4	1	71	7.0	36	1.65	53	19.47
2	1	1	0	65	8.0	40	1.75	70	22.86
3	2	5	1	70	7.0	39	1.65	60	22.04
4	1	5	1	30	6.5	42	1.73	59	19.71
5	1	4	1	45	7.5	39	1.75	80	26.12
6	1	5	1	62	7.0	40	1.70	60	20.76
7	2	5	1	74	6.0	37	1.60	50	19.53
8	1	3	1	34	6.0	32	0.88	25	32.28
9	1	5	1	70	8.0	38	1.70	60	20.76
10	1	5	1	61	8.0	38	1.70	80	27.68
11	1	2	0	63	7.5	43	1.79	80	25.07
12	1	2	0	48	7.5	40	1.75	65	21.22
13	1	2	0	81	6.5	39	1.70	55	19.03
14	1	2	0	73	7.0	45	1.78	85	26.83
15	1	1	0	70	8.0	41	1.65	60	22.04
16	2	3	1	65	7.0	45	1.96	41	10.64
17	1	5	1	47	7.0	44	1.77	70	22.34
18	1	3	1	86	8.0	42	1.72	85	28.73
19	2	1	0	88	5.5	35	1.58	40	16.02
20	1	4	1	47	6.0	44	1.78	68	21.46
21	1	2	0	46	7.0	46	1.70	150	51.90
22	1	4	1	75	6.0	38	1.63	65	24.46
23	1	1	0	32	6.5	40	1.78	69	21.78
24	2	4	1	83	6.5	39	1.56	50	20.55
25	1	5	1	83	6.5	38	1.66	70	25.40
26	1	1	0	64	6.0	43	1.74	80	26.34
27	2	1	0	46	7.5	41	1.66	80	28.99
28	1	2	0	69	7.5	43	1.70	80	27.54
29	2	5	1	55	6.9	45	1.50	100	44.20
30	1	2	0	64	7.2	43	1.77	80	25.67
31	1	2	0	45	6.1	36	1.60	43	16.67
32	1	5	1	86	7.0	38	1.68	66	23.38
33	2	3	1	93	7.0	35	1.91	85	23.40
34	2	3	1	83	7.0	35	1.53	83	35.38
35	1	3	1	63	7.0	35	1.52	97	42.12
36	1	4	1	75	7.0	35	1.51	81	35.59
37	1	4	1	75	6.7	36	1.51	42	18.49
38	1	4	1	82	6.4	35	1.87	61	17.41
39	1	4	1	56	6.0	45	1.50	45	19.86
40	1	4	1	62	7.0	35	1.50	85	37.69
41	2	4	1	52	7.0	43	1.54	59	24.67
42	1	5	1	61	7.0	35	1.50	89	39.56
43	1	5	1	76	7.0	35	1.93	42	11.14
44	1	5	1	69	6.5	35	1.72	100	33.75
45	1	5	1	73	6.0	45	1.50	45	19.85
46	2	5	1	15	6.2	45	1.50	44	19.29

For research review

47	1	5	1	44	6.7	45	1.51	44	19.41
48	1	1	0	38	7.0	45	1.72	70	23.66
49	2	5	1	75	7.0	42	1.50	140	62.22

For research review

GCS-M	AP-II	NSE-0h	NSE-48h	LOSd	Type	CauseCardiogenic=0；Cerebral=2；	pre-CPRtime 0＜4min 1＞4min	CPRtime 0＜30min；1＞30min	Initial-Rhythm ShR=0
3	23	NA	NA	3	IHCA	0	0	0	1
6	15	58.2	300	15	IHCA	0	0	0	0
2	21	NA	NA	7	OHCA	0	1	0	0
1	23	NA	NA	2	IHCA	2	0	0	0
4	21	NA	NA	1	OHCA	2	1	0	1
1	27	NA	NA	7	IHCA	2	0	0	1
2	28	NA	NA	1	OHCA	2	1	1	1
4	17	14.6	24.9	10	OHCA	1	0	0	1
1	25	NA	NA	3	IHCA	1	0	0	1
1	28	NA	NA	7	OHCA	2	1	1	1
6	23	NA	NA	9	IHCA	0	0	0	1
4	15	NA	NA	3	OHCA	1	1	0	0
4	13	47.5	NA	7	IHCA	0	0	1	0
5	13	42.9	NA	5	IHCA	0	0	0	1
5	13	19.2	19.2	15	IHCA	0	0	0	0
4	18	NA	NA	3	IHCA	0	1	1	1
2	21	NA	NA	10	OHCA	1	1	1	1
4	29	24.6	16.7	9	IHCA	0	0	0	1
4	19	NA	NA	7	IHCA	1	0	0	1
1	30	NA	NA	3	OHCA	0	1	1	1
3	15	NA	NA	19	IHCA	1	0	0	1
1	27	NA	NA	3	OHCA	0	0	1	1
4	30	57.3	30.1	21	IHCA	0	0	0	0
4	23	NA	NA	9	IHCA	2	0	0	1
1	30	NA	NA	3	IHCA	2	0	1	1
5	12	NA	NA	23	OHCA	1	1	1	1
3	11	NA	NA	14	IHCA	0	0	0	0
5	27	NA	NA	11	IHCA	0	0	0	0
1	27	NA	NA	3	IHCA	0	0	0	1
6	22	NA	NA	5	IHCA	0	0	0	1
3	13	NA	NA	7	IHCA	1	0	0	0
1	23	29	NA	3	IHCA	0	0	0	1
4	19	NA	NA	1	IHCA	0	0	0	1
4	26	NA	NA	1	IHCA	0	1	1	1
1	23	NA	NA	5	IHCA	0	0	0	1
1	17	NA	NA	5	IHCA	0	0	0	0
1	23	NA	NA	1	IHCA	1	0	0	1
1	27	NA	NA	7	IHCA	1	1	1	1
1	20	NA	NA	1	IHCA	2	1	1	0
1	28	NA	NA	1	IHCA	0	0	0	0
1	30	NA	NA	1	IHCA	0	0	0	1
2	31	NA	NA	1	IHCA	1	0	0	0
2	26	NA	NA	3	IHCA	0	0	0	1
1	27	NA	NA	1	IHCA	1	1	1	1
1	28	NA	NA	1	IHCA	2	0	1	1
4	27	NA	NA	4	IHCA	0	0	0	1

For research review

4	27	NA	NA	9	IHCA	0	1	0	1
6	29	27.7	11.7	9	IHCA	0	0	0	1
1	30	12.5	NA	1	IHCA	1	0	0	1

For research review

lac	pupil diameter mm	light reflex+1，-0	ONSD/H mm/m	ONSD/W mm/kg	ONSD-BMImm*m2/kg	ONSD/PDmm/cm	ONSD/S mm/cm	ONSD-6hmm	ONSD/ETD-6h
12.1	2.0	1	3.889	0.121	0.330	0.917	0.178	6.417	0.292
13.2	3.0	1	3.019	0.075	0.231	0.660	0.132	5.283	0.244
11.6	6.0	1	3.444	0.095	0.258	0.812	0.146	5.683	0.245
10.8	1.0	1	4.114	0.121	0.361	1.095	0.169	7.117	0.315
9.2	2.0	1	3.705	0.081	0.248	0.864	0.166	6.483	0.287
13.6	3.0	1	3.118	0.088	0.255	0.757	0.133	5.300	0.252
12.2	5.0	1	3.500	0.112	0.287	0.933	0.151	5.600	0.259
11.5	5.0	1	6.686	0.235	0.182	0.981	0.184	5.883	0.259
13.4	2.0	1	4.284	0.121	0.351	0.910	0.192	7.283	0.330
7.2	5.0	1	3.314	0.070	0.204	0.704	0.148	5.633	0.253
2.9	2.0	1	2.542	0.060	0.191	0.640	0.112	4.800	0.204
4.1	2.0	0	2.457	0.066	0.203	0.573	0.108	4.300	0.179
12.7	4.0	1	3.333	0.103	0.298	0.872	0.145	5.667	0.241
3.7	2.5	1	2.453	0.051	0.163	0.624	0.097	4.367	0.197
1.6	2.0	0	3.434	0.094	0.257	0.708	0.138	5.667	0.249
5.9	3.5	0	2.482	0.119	0.458	0.696	0.108	4.870	0.216
15.8	4.0	1	3.766	0.095	0.298	0.952	0.152	6.667	0.280
5.2	3.0	0	3.769	0.076	0.226	0.810	0.154	6.483	0.281
2.6	2.0	0	3.544	0.140	0.350	1.018	0.160	5.600	0.254
NA	4.0	1	4.082	0.107	0.339	1.211	0.165	7.267	0.290
2.2	3.0	0	3.403	0.039	0.111	0.826	0.126	5.785	0.253
NA	5.0	1	4.162	0.104	0.277	1.131	0.179	6.783	0.331
11.5	3.5	1	3.090	0.080	0.253	0.846	0.138	5.500	0.238
2.1	4.0	1	2.981	0.093	0.226	0.715	0.119	4.650	0.232
3	1.5	0	3.755	0.089	0.245	0.959	0.164	6.233	0.271
2.6	2.5	1	3.004	0.065	0.199	0.871	0.122	5.229	0.238
6.7	3.5	1	3.448	0.071	0.196	0.756	0.138	5.671	0.255
5.7	3.5	1	3.258	0.069	0.199	0.730	0.127	5.478	0.239
NA	1.5	0	4.610	0.070	0.157	1.006	0.154	6.920	0.323
25.5	2.0	1	2.800	0.063	0.196	0.704	0.117	5.035	0.251
7.6	4.0	1	3.608	0.137	0.350	0.954	0.162	5.837	0.268
8.6	1.0	1	3.919	0.100	0.282	0.940	0.173	6.583	0.281
12.1	5.0	0	3.076	0.069	0.250	0.837	0.167	5.860	0.263
11.9	3.0	0	3.867	0.071	0.167	0.846	0.169	5.920	0.262
15.4	4.0	0	4.091	0.064	0.147	0.887	0.177	6.210	0.280
9.1	4.5	0	4.120	0.077	0.175	0.890	0.178	6.230	0.282
13.4	1.0	0	4.346	0.156	0.354	0.982	0.182	6.550	0.291
8.9	5.5	1	3.200	0.098	0.344	0.939	0.171	5.990	0.266
10.6	4.5	0	4.737	0.159	0.359	1.187	0.158	7.120	0.311
15.7	5.5	0	4.231	0.075	0.168	0.907	0.181	6.350	0.263
2.8	2.5	0	3.761	0.099	0.235	0.829	0.135	5.800	0.258
9.8	1.5	0	4.133	0.070	0.157	0.886	0.177	6.200	0.274
8.9	3.5	1	2.699	0.125	0.469	0.746	0.149	5.220	0.230
12.2	5.0	0	3.161	0.054	0.161	0.837	0.155	5.440	0.245
3.4	6.0	0	4.720	0.159	0.357	1.180	0.157	7.080	0.315
NA	3.0	0	4.461	0.154	0.347	1.079	0.149	6.700	0.309

For research review

NA	1.0	0	4.379	0.150	0.339	0.986	0.146	6.590	0.287
25	4.0	0	3.149	0.077	0.229	0.774	0.120	5.417	0.222
11.6	4.5	1	4.311	0.046	0.104	0.924	0.154	6.467	0.307

For research review

ONSD-24hcm	ONSD/ETD-24h	ONSD-48hcm	ONSD/ETD-48h	CCAUnilateral MCA circulatory arrest	PSV-0hcm-s	EDV-0hcm-s	MV-0hcm-s	PI-0hcm-s	RI-0hcm-s
NA	NA	NA	NA	0	51.573	15.943	39.697	0.898	0.691
5.200	0.218	4.973	0.248	0	54.383	5.313	38.027	1.290	0.902
5.442	0.249	5.386	0.223	1	37.180	-9.390	21.657	2.150	1.253
7.113	0.322			0	127.180	65.190	85.850	0.722	0.612
6.467	0.283	NA	NA	1	63.767	33.447	53.660	0.565	0.475
5.256	0.262	5.159	0.261	0	50.320	13.923	38.188	0.953	0.723
5.600	0.258	NA	NA	1	58.327	-12.603	34.683	2.045	1.216
5.634	0.225	NA	NA	0	30.153	16.820	25.709	0.519	0.442
7.100	0.321	NA	NA	1	49.037	6.687	34.920	1.213	0.864
5.233	0.235	5.267	0.240	0	43.827	6.743	31.466	1.179	0.846
6.258	0.262	6.432	0.270	NA	NA	NA	NA	NA	NA
NA	NA	NA	NA	0	71.400	31.337	58.046	0.690	0.561
5.817	0.266	5.317	0.230	0	42.780	11.715	22.070	1.408	0.726
NA	NA	NA	NA	0	64.167	11.897	46.743	1.118	0.815
5.650	0.239	6.617	0.278	0	36.400	19.327	30.709	0.556	0.469
NA	NA	NA	NA	NA	NA	NA	NA	NA	NA
NA	NA	NA	NA	1	127.520	-25.060	25.800	5.914	1.197
6.450	0.293	6.567	0.291	0	78.960	23.187	41.778	1.335	0.706
5.367	0.240	5.367	0.232	0	56.640	28.140	47.140	0.605	0.503
7.267	0.290	7.226	0.311	NA	NA	NA	NA	NA	NA
5.615	0.253	5.709	0.259	NA	NA	NA	NA	NA	NA
6.698	0.311			NA	NA	NA	NA	NA	NA
6.480	0.274	6.503	5.386	0	58.230	24.050	35.443	0.964	0.587
NA	NA	NA	NA	NA	NA	NA	NA	NA	NA
NA	NA	NA	NA	NA	NA	NA	NA	NA	NA
6.016	0.265	NA	NA	NA	NA	NA	NA	NA	NA
5.532	0.248	5.500	0.268	NA	NA	NA	NA	NA	NA
5.290	0.236	NA	NA	NA	NA	NA	NA	NA	NA
NA	NA	NA	NA	NA	NA	NA	NA	NA	NA
5.048	0.243	4.974	0.221	NA	NA	NA	NA	NA	NA
5.774	0.258	5.709	0.257	NA	NA	NA	NA	NA	NA
NA	NA	NA	NA	NA	NA	NA	NA	NA	NA
NA	NA	NA	NA	NA	NA	NA	NA	NA	NA
NA	NA	NA	NA	NA	NA	NA	NA	NA	NA
NA	NA	NA	NA	NA	NA	NA	NA	NA	NA
NA	NA	NA	NA	NA	NA	NA	NA	NA	NA
NA	NA	NA	NA	NA	NA	NA	NA	NA	NA
NA	NA	NA	NA	NA	NA	NA	NA	NA	NA
7.081	0.298	NA	NA	NA	NA	NA	NA	NA	NA
NA	NA	NA	NA	NA	NA	NA	NA	NA	NA
NA	NA	NA	NA	NA	NA	NA	NA	NA	NA
NA	NA	NA	NA	NA	NA	NA	NA	NA	NA
NA	NA	NA	NA	NA	NA	NA	NA	NA	NA
NA	NA	NA	NA	NA	NA	NA	NA	NA	NA
7.182	0.292	NA	NA	NA	NA	NA	NA	NA	NA
NA	NA	NA	NA	NA	NA	NA	NA	NA	NA

For research review

NA	NA	NA	NA	NA	NA	NA	NA	NA	NA
5.417	0.222			0	107.620	24.787	52.398	1.581	0.770
NA	NA	NA	NA	NA	NA	NA	NA	NA	NA

For research review

S/D-0hcm-s	PSV-24hcm/s	EDV-24hcm/s	MV-24hcm/s	PI-24h	RI-24h	S/D-24h
3.235	NA	NA	NA	NA	NA	NA
10.235	59.387	10.313	43.029	1.140	0.826	5.758
-3.960	NA	NA	NA	NA	NA	NA
1.951	NA	NA	NA	NA	NA	NA
1.907	94.397	0.653	63.149	1.484	0.993	
3.614	NA	NA	NA	NA	NA	NA
-4.628	43.637	-17.190	23.361	2.604	1.394	-2.538
1.793	NA	NA	NA	NA	NA	NA
7.333	55.907	-12.300	33.171	2.056	1.220	-4.545
6.499	71.320	18.077	53.572	0.994	0.747	3.945
NA	NA	NA	NA	NA	NA	NA
2.278	NA	NA	NA	NA	NA	NA
3.652	NA	NA	NA	NA	NA	NA
5.394	87.733	24.440	66.636	0.950	0.721	3.590
1.883	48.143	17.507	37.931	0.808	0.636	2.750
NA	NA	NA	NA	NA	NA	NA
-5.089	NA	NA	NA	NA	NA	NA
3.405	126.413	47.760	73.978	1.063	0.622	2.647
2.013	NA	NA	NA	NA	NA	NA
NA	NA	NA	NA	NA	NA	NA
NA	NA	NA	NA	NA	NA	NA
NA	NA	NA	NA	NA	NA	NA
2.421	82.353	43.193	56.246	0.696	0.476	1.907
NA	NA	NA	NA	NA	NA	NA
NA	NA	NA	NA	NA	NA	NA
NA	NA	NA	NA	NA	NA	NA
NA	NA	NA	NA	NA	NA	NA
NA	NA	NA	NA	NA	NA	NA
NA	NA	NA	NA	NA	NA	NA
NA	NA	NA	NA	NA	NA	NA
NA	NA	NA	NA	NA	NA	NA
NA	NA	NA	NA	NA	NA	NA
NA	NA	NA	NA	NA	NA	NA
NA	NA	NA	NA	NA	NA	NA
NA	NA	NA	NA	NA	NA	NA
NA	NA	NA	NA	NA	NA	NA
NA	NA	NA	NA	NA	NA	NA
NA	NA	NA	NA	NA	NA	NA
NA	NA	NA	NA	NA	NA	NA
NA	NA	NA	NA	NA	NA	NA
NA	NA	NA	NA	NA	NA	NA
NA	NA	NA	NA	NA	NA	NA
NA	NA	NA	NA	NA	NA	NA
NA	NA	NA	NA	NA	NA	NA
NA	NA	NA	NA	NA	NA	NA
NA	NA	NA	NA	NA	NA	NA

For research review

NA	NA	NA	NA	NA	NA	NA
4.342	NA	NA	NA	NA	NA	NA
NA	NA	NA	NA	NA	NA	NA

For research review
